# The Role and Clinical Relevance of Disseminated Tumor Cells in Breast Cancer

**DOI:** 10.3390/cancers6010143

**Published:** 2014-01-15

**Authors:** Malgorzata Banys, Natalia Krawczyk, Tanja Fehm

**Affiliations:** 1 Department of Obstetrics and Gynecology, University of Duesseldorf, Duesseldorf D-40225, Germany; E-Mails: natalia_krawczyk@yahoo.de (N.K.); tanja.fehm@t-online.de (T.F.); 2 Department of Obstetrics and Gynecology, Marienkrankenhaus Hamburg, Hamburg D-22087, Germany

**Keywords:** breast cancer, bone marrow, disseminated tumor cell, circulating tumor cell, tumor cell dissemination

## Abstract

Tumor cell dissemination is a common phenomenon observed in most cancers of epithelial origin. One-third of breast cancer patients present with disseminated tumor cells (DTCs) in bone marrow at time of diagnosis; these patients, as well as patients with persistent DTCs, have significantly worse clinical outcome than DTC-negative patients. Since DTC phenotype may differ from the primary tumor with regard to ER and HER2 status, reevaluation of predictive markers on DTCs may optimize treatment choices. In the present review, we report on the clinical relevance of DTC detection in breast cancer.

## 1. Introduction

The speculations on dissemination patterns of solid malignancies and the role of microenvironment in disease progression were made by several researchers in 19th century [1,2]. The presence of minimal residual disease (MRD) may influence the patient's prognosis despite successful tumor excision and completed adjuvant therapy. Isolated tumor cells can be searched for in two main compartments; cancer cells detected in peripheral blood are described as circulating tumor cells (CTCs), while those in the bone marrow are called disseminated tumor cells (DTCs). While the major part of current translational research focuses on detection of CTCs in the blood, the first valid prognostic data on minimal residual disease in breast cancer were provided by studies on DTCs in bone marrow. The aim of this review is to give an account of current findings on tumor cell dissemination into bone marrow in breast cancer patients, with respect to the features of DTCs, their clinical impact and future possibilities. 

## 2. Methods for Detection of DTCs

Numerous protocols have been established for the isolation of DTCs from bone marrow and CTCs from peripheral blood. Most authors perform bone marrow aspiration from one or both iliac crests using Jamshidi’s biopsy technique [3]. The concentration of DTCs in the bone marrow is low, estimated at one tumor cell per 10^7^–10^8^ blood cells in patients with advanced cancer; therefore, some protocols contain an enrichment step [4,5]: (1) density gradient centrifugation where mononuclear cells are separated from other blood cells; (2) positive selection where tumor cells are enriched through the use of an antibody targeted against a tumor cell marker (e.g., cytokeratins, EpCAM) or (3) negative selection where the antibody is targeted against a leukocyte antigen (e.g., CD45). For the detection of tumor cells, both antibody-based methods and molecular assays are in use. Methods for isolating DTCs/CTCs and criteria for their classification have been described in detail by Fehm *et al.* [6].

### 2.1. Antibody-Based Detection

The most widely used and standardized detection systems are antibody-based immuno¬cytochemistry and immunofluorescence; they rely on the capture of cells expressing antigens absent from other blood cells, e.g., epithelial (e.g., cytokeratin, EpCAM) [7] or breast tissue markers (e.g., human mammaglobin). Genotyping studies have provided evidence that cells detected by cytokeratin staining are in fact malignant [8]. Both the staining pattern and morphological criteria can be analyzed. Phenotypic features of tumor cells have been listed in the consensus recommendations for DTC detection [5]. Both immunocytochemistry and fluorescence enable direct quantification of tumor cells. Automated analyzers facilitate the screening process and enable cytologists to analyze more samples in a given time [9]. 

### 2.2. Molecular Detection

Molecular assays target epithelial or tissue-specific mRNA [6,10]. Common markers include various cytokeratins, EpCAM and mammaglobin. RT-PCR based systems are extremely sensitive; however, false positive results may occur due to illegitimate transcription of pseudogenes, and low-level transcription of markers present on cells other than tumor cells. RT-PCR based methods frequently establish a cutoff value to differentiate between positive and negative results. An advantage of multiplex PCR is the evaluation of many different markers at the same time [11].

### 2.3. Commercially Available Assays

Some of the established detection protocols are commercially available. The most widely used assay, the CellSearch system (Veridex, Warren, NJ, USA), has been approved by the FDA for the detection of CTCs in metastatic breast, prostate and colorectal cancer, and has been used for tumor cell detection in translational research programs within large clinical trials. The CellSearch system is a semiautomated antibody-based quantitative technique based on immunofluorescence and flow cytometry [12,13]. CTCs are enriched by immunomagnetic beads linked with anti-EpCAM antibodies and identified by cytokeratin-positivity, positive nuclear staining and CD45 negativity. The AdnaTest BreastCancer (AdnaGen AG, Langenhagen, Germany) is a commercially available molecular assay. CTCs are enriched by immunomagnetic beads labeled with anti-MUC1 and anti-EpCAM antibodies and detected by multiplex RT-PCR based on three markers (GA 73.3, EpCAM and HER2) [11,14,15]. The concordance rate between CellSearch and AdnaTest is relatively high (70%–90%) [11]. 

## 3. Clinical Relevance of DTC Detection in Bone Marrow

### 3.1. Prognostication

Despite remarkable improvements in oncological diagnosis and treatment, 25% to 30% of primary breast cancer patients may suffer from disease recurrence years after primary diagnosis [16]. Since distant relapse is diagnosed in a significant proportion of patients without lymph node involvement who underwent complete surgical removal of the primary tumor, identification and evaluation of new prognostic factors predicting unfavourable clinical outcome have become a major focus of translational oncologic research in the past two decades. 

Continuous spread of cancer cells into blood vessels and its role in metastatic cascade have been already described by studies originated in the 19th century [1,2]. In 1889, Paget suggested in his “seed and soil” hypothesis that interactions between spread tumor cells and microenvironment of secondary homing sites may potentially lead to development of distant metastases [2]. Based on animal models, thousands of epithelial tumor cells disseminate daily into blood circulation; most of these cells have very short lifespan, some are already apoptotic or dead, while others are assumed to be eliminated by shear forces of the bloodstream [17,18]. However, in up to 30% of patients tumor cells are able to persist in the blood circulation after extirpation of primary tumor possibly leading to the late relapse of disease [19,20]. 

Tumor cell dissemination into bone marrow (BM) can be observed in 30%–40% of primary breast cancer patients [7]. Prognostic significance of DTCs in BM was reported by several researchers [21,22,23,24]. In 2005, Braun *et al.* confirmed in a large pooled analysis of more than 4,700 patients with primary breast cancer that DTC detection in BM at the time of primary diagnosis independently predicts unfavourable clinical outcome (Level 1 evidence) [7]. Moreover, as demonstrated by numerous studies, DTCs are able to persist in BM after completion of adjuvant treatment. These persistent DTCs were also shown to be of negative prognostic value [25,26]. Hartkopf *et al.* demonstrated that persistence of DTCs after systemic treatment is a strong and independent marker of reduced disease-free and overall survival [27]. Thus, presence of isolated tumor cells in BM of breast cancer patients is regarded as a surrogate marker of minimal residual disease. 

Although haematogenous tumor spread may indicate early generalisation of disease, only 40%–60% of breast cancer patients with DTCs in BM will suffer from a relapse [7,23]. According to the hypothesis of “metastatic inefficiency”, only a fraction of tumor cells is able to survive at the secondary sites and cause tumor growth [28,29]. Factors determining if single tumor cells form micro- and macro-metastases at distant sites remain yet to be clarified. Therefore, beyond mere detection of these cells, their further characterization is gaining clinical importance. 

### 3.2. Characterization of DTCs

Eradication of minimal residual disease has become a desired aim of every systemic breast cancer treatment; DTCs, as a surrogate marker of MRD, represent therefore potential targets for future adjuvant therapies. Major efforts have been made to characterize these cells with regard to their pheno- and genotype. Pantel *et al.* demonstrated that DTCs are distinguished by a low expression of proliferative markers, such as Ki-67 or p120 [30]. This phenomenon could possibly explain their ability to survive antiproliferative cytotoxic treatment [26,31]. It supports also the theory of cancer cell dormancy; this hypothesis holds that isolated tumor cells at secondary sites are able survive in a quiescent state, either withdrawing completely from the cell cycle or persisting at a slow proliferation rate counterbalanced by apoptosis [32]. Furthermore, the surface of DTCs is characterized by reduced expression of MHC class I molecules, which could result in an immune escape of these cells [33], as well as high activity of tumor invasiveness markers, such as urokinase plasminogen activator (uPA) and its membrane receptor (uPA-R), extracellular matrix metalloproteinase inducer *(EMMPRIN)* or cathepsin B, which enables destruction of basement membrane and stromal invasion [34,35,36,37]. Elevated expression of cell adhesion molecule E-cadherin on DTCs might be involved in the process of forming a metastasis [38]. 

Phenotypic characteristics of potential clinical interest in DTCs are the HER2 status and hormone receptor status. To date, several studies investigated the expression of these predictive markers on DTCs. Major phenotypic discrepancies between DTCs and primary tumor have been reported [30,39,40,41,42] (Table 1). Solomayer *et al.* investigated 137 primary breast cancer patients with regard to the HER2 status of DTCs; 38% of patients with HER2 negative breast cancer presented with HER2 positive DTCs [39]. Up to 87% of breast cancer patients have HER2 positive DTCs in BM whereas HER2 positivity rate of primary tumor reaches in average 15%–30% [30,43,44]. Previously, we reported a high rate of HER2-positive persistent DTCs in patients with HER2 negative primary tumor after completion of adjuvant therapy [42]. Since HER2 targeted therapy (e.g., trastuzumab, lapatinib) is intended only for patients with HER2 positive primary tumor, HER2 positive MRD in patients with HER2 negative primary lesion might elude systemic treatment.

The influence of HER2 targeted therapy on MRD has been recently evaluated by several authors. In the study by Bernhard *et al.* HER2-specific T-lymphocytes have been transferred to a patient with HER2 positive metastatic breast cancer leading to elimination of HER2 positive DTCs from the BM but not the solid metastases [45]. In the trial by Rack *et al.* ten primary breast cancer patients with persistent HER2-positive DTCs received trastuzumab therapy for 12 months; DTC status was then revaluated by follow-up BM biopsies at regular time intervals [46]. HER2 positive DTCs could be eradicated in all ten patients. However, clinical significance of MRD elimination remains unclear. Two randomized clinical trials, DETECT III and Treat CTC, have been initiated recently to evaluate whether patients with persistent isolated tumor cells benefit from HER2 targeted therapy based on HER2 status of their MRD [47,48].

**Table 1:** Expression profiles of DTCs in primary breast cancer patients.

Author	N	Method	Marker	Marker-positive DTCs	Discrepancy DTC ^1^/PT ^2^
Hartkopf *et al.*, 2013 [49]	151	ICC^3^	HER2	52%	49%
Fehm *et al.*, 2008 [41]	107	IFC^4^	ERα	12%	72%
Solomayer *et al*., 2006 [39]	46	IFC	HER2	43%	38%
Reimers *et al.*, 2004 [34]	11	ICC	EMMPRIN	89%	n. a.
Ditsch *et al.*, 2003 [40]	17	ICC	ERα	12%	53%
Braun *et al.*, 2001 [43]	52	ICC	HER2	60%	58%
Tögel *et al*., 2001 [37]	15	ICC	uPA-R	67%	n. a.
Braun *et al.*, 1999 [50]	15	ICC	HER2	87%	n. a.
Solomayer *et al.*, 1998 [35]	290	ICC	Catepsin D	9%	n. a.
Solomayer *et al.*, 1997 [36]	280	ICC	uPA	35%	n. a.
Funke *et al*., 1996 [38]	21	ICC	E Cadherin	71%	n. a.
Pantel *et al.*, 1993 [30]	69	ICC	P120/Ki67	16%	n. a.
Pantel *et al.*, 1993 [30]	71	ICC	HER2	68%	n. a.
Pantel *et al*., 1991 [33]	26	ICC	MHC I	50%	n. a.

n.a.: not analyzed, ^1^ DTC: Disseminated tumor cell(s); ^2^ PT: Primary tumor; ^3^ ICC: Immunocytochemistry; ^4^ IFC: Immunofluorescence.

Major phenotypic differences between primary tumor and DTCs have been reported with regard to hormone receptor status as well. DTCs are generally hormone receptor negative despite the hormone receptor positive primary tumor [20,40,51]. We reported previously on the ER status of DTCs in 107 primary breast cancer patients. Only 12 of 88 patients (14%) with ER positive primary tumor presented with ER positive DTCs in BM while the majority (86%) had ER negative DTCs [41]. This discrepancy may explain the failure of endocrine therapy in a subset of ER positive patients. 

### 3.3. Therapy Monitoring

At present, response to therapy is assessed using clinical examination, imaging and tumor markers. However, traditional restaging assays may be insufficient for predicting relapse/progression in asymptomatic “disease-free” patients. A significant percentage of patients who undergo adjuvant therapy have no evidence of the disease but will nonetheless suffer from subsequent metastasis years after completion of treatment. The effectiveness of the chosen treatment regime remains thus uncertain till evident relapse. Minimal residual disease is the only parameter available for evaluation after the primary tumor has been removed. Since the accessibility of assays based on bone marrow punctions is limited due to its invasiveness and costs, the majority of studies on therapy monitoring use CTC measurements in peripheral blood.

Several authors showed that changes in CTC levels enable prediction of treatment efficacy [13,47,52]. Smith *et al*. observed clinical changes mirrored by changes in CTC load in 68% of chemotherapy cycles [52]. A large study of Cristofanilli *et al*. proved elevated levels of CTC to be even more predictive than traditional assays (based on Response Evaluation Criteria in Solid Tumors) in metastatic breast cancer patients before start of first-line therapy [13]. Changes in CTC levels are not only associated with disease activity but may also precede clinical evidence of the progression/stabilization [53]. CTC detection might help in evaluating individual risk of relapse and thus identifying patients in need of additional (targeted) therapy.

In case of metastatic breast cancer, radiologic assays enable accurate assessment of tumor size during therapy but do not give insight into molecular changes of the disease. Multiple invasive biopsies of metastases are not routinely undertaken. Evaluation of predictive markers on CTCs during disease progression might be considered a “real-time biopsy” or “liquid biopsy” of metastasis and improve treatment decisions.

## 4. Conclusions

Single tumor cells in bone marrow and blood serve as surrogate parameter for minimal residual disease; detection and characterization of MRD may become an important tool in cancer diagnostics. Although the first valid data on MRD in breast cancer have been provided by studies evaluating DTCs in bone marrow, current research programs have focused on CTCs in peripheral blood. Potential areas for clinical implementation of MRD detection are therapy monitoring and optimization of treatment decisions based on characteristics of CTCs/DTCs.
